# A pragmatic suggestion for dealing with results for candidate genes obtained from genome wide association studies

**DOI:** 10.1186/1471-2156-8-20

**Published:** 2007-05-10

**Authors:** David Curtis, Anna E Vine, Jo Knight

**Affiliations:** 1 Centre for Psychiatry, Queen Mary's School of Medicine and Dentistry, London E1 1BB, UK; 2 Social Genetic & Developmental Psychiatry Centre, Institute of Psychiatry, De Crespigny Park, London SE5 8AF, UK

## Abstract

**Background:**

Researchers may embark on a genome-wide association study before fully investigating candidate regions which have been reported to produce evidence to suggest that they harbour susceptibility loci. If the genome wide study had not been carried out then results which demonstrated only modest statistical significance from candidate regions would be judged to be of interest and would stimulate further investigation. However if hundreds of thousands of markers are typed then inevitably very large numbers of such results will occur by chance and those from candidate regions may attract no special attention.

**Results:**

An approach is proposed in which differential treatment is afforded to markers from candidate regions and from those that are routinely typed in the context of a genome wide scan. Different prior probabilities are assigned to the two types of marker. A likelihood ratio is derived from the reported *p *value for each marker, calculated as LR = e^chiinv(1,p)/2^, and the posterior odds in favour of a true positive association are obtained. These odds can be used to rank the markers with a view to suggesting the regions in which further genotyping is indicated. We suggest that prior probabilities be specified such that a candidate marker significant at *p *= 0.01 and a routine marker significant at *p *= 0.00001 will yield similar values for the posterior odds. We show that this can be achieved by setting a value for prior probability of association to 0.1 for candidate markers and to 0.00018 for routine markers.

**Conclusion:**

It is essential that formal procedures be adopted in order to avoid modestly positively results from candidate regions being swamped by the huge number of nominally significant results which will be obtained when very many markers are genotyped. Software to carry out the conversion from *p *values to posterior odds is available from .

## Background

The ability to carry out so-called genome wide association studies using a standard panel of single nucleotide polymorphisms (SNPs) presents obvious difficulties. For many diseases it will be the case that particular genes have already been implicated as possibly or probably being involved. Typically, there will be some positive and some negative association studies, some groups will report positive results with particular markers or haplotypes while others will report negative results with those markers but positive results from some other markers nearby and a third set of groups will report positive results with a different, though related, phenotype. There may be general consensus that the gene is worthy of further investigation. In an ideal world groups possessing appropriate datasets would test additional polymorphisms within the gene in an attempt to provide some definitive answer as to whether or not it influences susceptibility. However, the world is not ideal and instead what may happen is that groups who have yet to fully investigate the gene may submit their datasets for genotyping with a standard, very large, set of SNPs which will include a few markers located in the gene in question along with a few hundred thousand which are not. The problem is that markers in the candidate gene may produce relatively modest evidence in favour of association, perhaps at the level of *p *< 0.01 or so, even with hundreds of subjects. If one were to study a candidate gene on its own and obtain results of this nature one might declare them of interest and as supporting further investigation. However if a genome wide scan of 500 K markers is performed then one can expect 5,000 to be significant at *p *< 0.01 and the evidence supporting the candidate gene will look very unimpressive indeed.

We should emphasise at this point that a marker having a real, but modest, effect is not expected to produce a smaller *p *value than markers producing apparently significant results by chance. If the expected significance, based on effect size and sample size, for a given truly associated marker is 0.01 then the *p *value actually obtained will probably be fairly close to this value. However among 500 K markers one may expect 50 or so to be significant at *p *< 0.0001 by chance and there is no reason to suppose that any of these will represent a true positive effect. If there are particular markers which are incorporated in the genome scan which do happen to be strongly associated with the disease then it is true that they may produce very highly significant *p *values. However our concern is that markers which are truly associated to some degree may yield only modest *p *values [1,2] and that their effect may be swamped if they are considered alongside the large number of other markers which are genotyped.

We propose that formal methods should be used for treating markers in candidate regions which have been previously implicated in a different fashion from the many thousands of SNPs spread across the genome which will be routinely typed in the course of a genome scan. One approach which would be theoretically sound would be to declare in advance a set of genes, and hence markers, which one regarded as being worthy of special consideration and to go on to analyse them first, before giving any consideration to those routinely genotyped. One could go on to publish the results from these analyses before analysing the other markers. However we believe that in practice it would be difficult to implement this approach. Typically, when a genome scan is performed all markers are analysed at once. It would be difficult to persuade readers and reviewers that special attention should be paid to certain markers giving results at *p *< 0.01 when one has the results for 5,000 more which are just as significant.

One approach which is relevant in this context is to estimate the false positive report probability (FPRP) [3]. This aims to use the prior probability for a marker to be associated in order to come up with a threshold *p *value such that markers achieving that threshold will have less than the declared probability of being false positive. Guidelines have been proposed in which candidate genes and genome screens can be treated differently when the FPRP approach is applied in order to satisfy different criteria for a finding to be "considered noteworthy" [4]. However these guidelines did not explicitly tackle the issue of dealing with candidate gene polymorphisms genotyped within the context of a genome wide association study. An alternative suggestion was to carry out weighting using the results of previous linkage studies [5]. This used a quantitative score in favour of linkage as a weighting factor, rather than declaring particular genes or markers as being *a priori *of interest.

We believe that the FPRP approach is not quite correct from a conceptual point of view when assessing the results of genome wide association studies. One reason is that such a study must always be regarded as an intermediate step. This is because the polymorphisms which are genotyped have been selected on the grounds of being appropriately spaced or because they tag other markers but they are not themselves expected to directly influence susceptibility to a disease. Rather, it is hoped that a genotyped marker showing association with the phenotype may be in linkage disequilibrium with a polymorphism which does influence susceptibility. Thus a positive finding should lead on to further genotyping of other polymorphisms in the region. In this situation, there is no point in fretting over what the probability is that one has a "noteworthy" finding. Once the scan has been performed one will inevitably want to go on to perform further genotyping and the question will be which regions appear the most promising to pursue. Thus the aim of the scan is to assist in the ranking of regions for further genotyping rather than to come up with any definitive answers as to whether particular regions are implicated or not. Once reasonable attempts have been made to type all available polymorphisms within a region then one can address the question of the overall strength of evidence in favour of association. One can then make a judgement as to whether it is worth proceeding to more demanding investigations such as functional studies. At this point one may feel that a method which explicitly aims to quantify the probability that a finding represents a true positive is of value. However we do not believe that such judgements are necessary when the question is not whether, but where, to perform further genotyping. Additional disadvantages of the FPRP approach are that one needs to declare the power of each test, that is the probability that a marker near a susceptibility locus will support association at a certain significance level, and that one must declare a plausible prior probability for each marker being truly associated. It is doubtful whether either of these values can be realistically quantified but the application of FPRP seems to yield an artificially concrete estimate of a probability which is in reality quite uncertain.

An alternative approach using a Bayesian strategy has been proposed which uses the prior probability for a test to detect association in order to weight the *p *value obtained [6]. The weighting scheme given as an example uses the number of potentially pathogenic polymorphisms detected by each tagging marker or group of markers to accord more weight to more informative markers although other weighting schemes are suggested, for example considering whether or not an SNP is coding or lies under a linkage peak. Deriving the necessary weights is mathematically relatively complex compared to the simple scheme we describe below.

Here we propose a related method which pragmatically seeks to rank results in a comparative way rather than to come up with absolute judgements as to whether they reflect true or false positive results. It also explicitly aims to afford special treatment for already implicated candidate genes within the context of a genome wide association study. Although it utilises "prior probabilities" for association of markers within candidate genes and of routinely typed markers it is recognised that these probabilities are essentially arbitrary and they are used mainly as a way of distinguishing the two sets of markers. It does not require any assumptions about the genetic effect size or power to detect association.

## Results

The procedure we arrive at consists of a number of stages. The first, and perhaps most problematic, step is to divide the markers into those which are candidate markers and those which are routinely genotyped. Candidate markers must either all be declared in advance or must be definable by some explicit rule. They must not have been already typed in the current sample. (Markers which have not been previously typed but which are in linkage disequilibrium with markers which have produced positive results in the current sample must be treated as a special case.) The authors reporting the study must report the basis on which they have declared markers as candidates. An example of a rule for defining a candidate marker might be to say that one will include all markers within 200 kb of a gene which has shown evidence from either three studies at p < 0.01, two at 0.001 or one at 0.0001 and for which the ratio of negative to positive association studies does not exceed 2:1. We believe that such a rule might reasonably reflect how association studies are commonly interpreted but we emphasise that any rule can be chosen, as long as it is explicit. Of course, one could set up hierarchies of candidate regions, whereby some were regarded as more strongly supported than others, but given the difficulty in making firm judgements about the weight of evidence any particular study provides we doubt that such complex schemes would be justified.

Next, appropriate values for the prior probabilities, and hence odds, of association for candidate and routinely typed markers should be chosen. From the argument set out in the *Methods *section we would suggest prior probabilities of PPr_CAND _= 0.1 and PPr_ROUT _= 0.00018 but obviously these values are to a large extent arbitrary.

Finally, for each marker and the *p *value we obtain for it we write:

OPo = OPr* e^chiinv(p,1)/2^

We can use either OPr_CAND _= PPr_CAND_/(1-PPr_CAND_) or OPr_ROUT _= PPr_ROUT_/(1-PPr_ROUT_) as the prior odds depending on how we have categorised the marker and we can then go ahead to rank all results according to the posterior odds of association. If we wish to obtain the posterior probability rather than odds we can write ProbPo = OPo/(OPo+1), which for the example above is equal to 0.755 for either the candidate marker significant at 0.01 or the routine marker significant at 0.00001. However we again emphasise that we do not regard the posterior odds or probability as representing an absolute value but rather as acting as a means to rank the results from different markers in order to guide further investigation.

## Conclusion

The main emphasis of our proposal is that approaches are adopted which provide differential treatment to markers in candidate genes and those which are routinely genotyped within a genome wide association study. Although we do not think the differences are critically important, our procedure does involve a slightly different emphasis from the FPRP approach. In order to obtain a value for the FPRP one must declare the power of the test to detect association. We believe it is fair to say that in most circumstances, at least within the context of markers typed routinely in the context of a genome scan, one can say nothing about the minimum value for the true genetic effect. Yet if the power of the test is declared to be higher than it actually is then the FPRP value will be too low. Ultimately, if the true genetic effect is minimal and the power to detect it is close to zero then the actual probability that one has a false positive finding will be close to one – all positives will be false positives. Yet a value for the FPRP based on some over-optimistic assessment of power may be quite small. We believe that in practice the value calculated for the FPRP may well not reflect the true probability that a finding is a false positive.

The alternative approach which we suggest does not require that any value for the power be specified. Rather, our method explicitly use a "best case" scenario. Rather than take the likelihood for the observations conditional on a pre-specified alternative hypothesis, our approach considers the alternative hypothesis which provides the best fit to the data, the maximum likelihood hypothesis. The criticism of this approach would be that it does not represent a true Bayesian scenario in which explicit statements are made about the prior probabilities of alternative hypotheses and the associated likelihoods of the observed data. The advantage of the approach is that it is not sensitive to any user-defined values regarding the genetic model, linkage disequilibrium parameters, sample size and associated power. It always explicitly deals with the alternative hypothesis which, *post hoc*, is determined to best fit the data. We have emphasised throughout that we would not regard the posterior odds or probability arising from this approach as truly reflecting the actual chances that a locus is involved in susceptibility. We believe that the FPRP value is in more danger of such a literal interpretation. In order to avoid such a literal interpretation we suggest that in applying our approach it may be preferable to quote the posterior "odds in favour of association" rather than "probability of association". That said, we do offer another approach to interpreting the posterior odds or probability obtained from our procedure. We suggest that it might be explicitly recognised as representing a best case scenario. Thus, if we were obtain a posterior probability of association of 0.75 we might make a statement along the lines of "the probability that this result reflects a true positive association is no more than 0.75". Arguing along these lines, we could say that the FPRP approach seeks to declare an estimate for the probability that a result is a false positive whereas ours seeks to declare a minimum probability for a result being a false positive, with fewer prior assumptions. We emphasise that we do not regard this distinction as critical.

What we do regard as being of critical importance is to have some formal, public system for treating results from candidate regions differently from routinely typed markers within the context of whole genome scans. Whether our method or FPRP or some related procedure is used, it is essential that markers from candidate regions can be treated as special cases. If this is not done then important findings from such markers will be swamped. Large samples will typically have been collected over a number of years at considerable effort. Once they have been genotyped for hundreds of thousands of markers they will be at risk as being regarded as "burned out". Any subsequent studies carried out on these samples yielding modest *p *values will be overshadowed by the huge numbers of genotypes already obtained. There is a sense that researchers submitting their painstakingly acquired datasets for genome-wide studies are sleep-walking into a desolate space from which nothing will emerge but a set of *p *values conforming pretty much to chance expectation. The approach we propose aims to mitigate some of the worst effects of this.

We note that our proposed procedure will do nothing to highlight the importance of true positive markers which yield modest *p *values but which are not yet regarded as candidates. Once genome scans have been completed and their results reported it will become necessary to review findings retrospectively from markers which "become interesting" after the scan is complete.

Doubtless these issues will be hotly debated, but we hope that the scheme we propose may represent a useful contribution to this debate and we look forward to the issues being progressed further.

## Methods

The situation we envisage is as follows. We have a dataset suitable for association studies containing perhaps several hundred cases and controls. We are about to submit the sample for a genome wide association scan using, say, 500 K SNPs. For the phenotype under consideration there exist a few genes for which there is quite strong, but not completely compelling, evidence for association. Alternatively, there may be genes which are very strong candidates for other reasons such as the results of expression studies or pathway analysis. If we were not performing the genome wide scan we would be typing additional polymorphisms in these genes. As it is, there are some 40–50 SNPs in these genes which will be typed in the genome wide study which have not already been typed. If we were typing these SNPs in isolation then we would take a result significant at *p *< 0.01 as being quite interesting and as supporting further investigation of this gene, for example by finding and typing additional polymorphisms within it. We realise that there are many theoretical problems with taking such an approach but we believe that it reasonably reflects the interpretation which in practice many investigators apply when assessing the results of association studies. We know that we expect 5 of the 500 K markers to be significant at *p *< 0.00001 purely by chance. It is also possible that some highly significant results from routinely typed markers may represent true genetic effects from hitherto unsuspected regions. We propose that we would like to achieve a scheme of ranking results such that a *p *value of 0.01 from a candidate marker will be considered similarly to a *p *value of 0.00001 from a routinely typed marker in terms of providing an indication of where further efforts may directed. We recognise that some genuinely associated markers may produce even smaller *p *values than this and they will receive favourable consideration whether or not they are recognised in advance as occurring in candidate genes.

All of this fits very comfortably into a Bayesian framework. Taking a relatively sceptical view we can say that if a gene has been the subject of a few positive and some negative association studies then the probability that that gene really does influence susceptibility and that a previously untyped polymorphism within it demonstrates association might be in the region of 0.1. Then we would say that the prior odds for a true positive association are 0.1/0.9. Then we could we carry out our genome wide association study and obtain the likelihood ratio for the results for a candidate polymorphism to be observed assuming association compared with the null hypothesis of no association. To apply a true Bayesian approach we would need to be able to calculate a Bayes factor consisting of the ratio of the cumulative probability distributions for the observed results under the alternative and null hypotheses. Instead, we propose a modified approach which uses instead the likelihood ratio obtained in the course of carrying out a likelihood ratio test for heterogeneity of allele frequencies between cases and controls. This test uses the two likelihoods maximised over allele frequencies under the alternative and null hypotheses. We use these maximised likelihoods as proxies for the distribution of likelihoods which might be obtained over the universe of alternative and null hypotheses and which would be needed in a true Bayesian approach. Multiplying this likelihood ratio by the prior odds provides an indication of the posterior odds for the polymorphism to be associated with the disease. We can do the same thing for a routinely typed polymorphism but assign it a lower prior probability of being truly associated. Writing OPr and OPo for prior and posterior odds and CAND and ROUT for a candidate or routine polymorphism, we have:

OPo_CAND _= OPr_CAND_*LR_CAND_

OPo_ROUT _= OPr_ROUT_*LR_ROUT_

Our task would then be to select suitable prior odds for association for a routinely typed marker such that a candidate marker with a likelihood ratio yielding a *p *value of 0.01 would yield similar posterior odds to a routine marker yielding a *p *value of 0.00001.

It is helpful to realise that, if one considers allele-wise tests for association with biallelic markers, there is a simple relationship between the *p *value and the likelihood ratio. Although in practice the test for association used may consist of a Pearson chi-squared statistic derived from a 2 × 2 contingency table it is safe to assume that this will be very similar to a likelihood ratio statistic calculated as 2*ln(LR) formulated as test for heterogeneity of allele frequencies between cases and controls. In the context of a genome wide association study this obviates the need to go through all the genotypings recalculating likelihood ratios because we can instead use the *p *values output from the analysis and convert each into the associated likelihood ratio. We can write *p *= chidist(*x*,*d*) to indicate the *p *value associated with a chi-squared statistic of *x *having *d *degrees of freedom and likewise we can write the inverse function *x *= chiinv(*p*,*d*) to indicate the chi-squared statistic, *x*, which would yield the stated *p *value. We can use this to select appropriate values for the prior odds for candidate markers and routine markers by considering the *p *values which we wish to make equivalent to each other. The allele-wise test for association yields a chi-squared test with one degree of freedom, so that we can state that a significance value of *p *= 0.01 from a candidate marker is equivalent to a chi-squared of chiinv(0.01,1) = 6.65 and a likelihood ratio of e^6.65/2 ^= 27.8, while a significance value of *p *= 0.00001 from a routine marker is equivalent to a chi-squared of chiinv(0.00001,1) = 19.5 and a likelihood ratio of e^19.5/2 ^= 17150. If our aim is to make both sets of results yield similar posterior odds for association, and if we assume prior odds for the candidate marker of 0.1/0.9 then we can write:

OPr_ROUT _= OPr_CAND _* LR_CAND_/LR_ROUT_

= 0.1/0.9 * 27.8/17150

= 0.00018

As in this case the odds are small they are practically equal to the probability, so using this scheme we would be declaring that a marker routinely genotyped as part of a genome wide association study has a prior probability of 0.00018, or approximately 1 in 5,000, of being truly associated. If one considers that there are 25,000 or so human genes and that several may be involved in the susceptibility to a particular disease then this estimate may not be terribly wide of the mark.

## Availability and requirements

Software to carry out the conversion from *p *values to posterior odds is available from . It is provided in the form of an Excel spreadsheet and also as the C source and MSDOS executable for a program which will read in output from the PLINK program, such as might be available from a genome wide association study.

## References

[B1] Farrall M, Morris AP (2005). Gearing up for genome-wide gene-association studies. Hum Mol Genet.

[B2] Ioannidis JP, Trikalinos TA, Khoury MJ (2006). Implications of small effect sizes of individual genetic variants on the design and interpretation of genetic association studies of complex diseases. Am J Epidemiol.

[B3] Wacholder S, Chanock S, Garcia-Closas M, El Ghormli L, Rothman N (2004). Assessing the probability that a positive report is false: an approach for molecular epidemiology studies. J Natl Cancer Inst.

[B4] Freimer NB, Sabatti C (2005). Guidelines for association studies in Human Molecular Genetics. Hum Mol Genet.

[B5] Roeder K, Bacanu SA, Wasserman L, Devlin B (2006). Using linkage genome scans to improve power of association in genome scans. Am J Hum Genet.

[B6] Pe'er I, de Bakker PI, Maller J, Yelensky R, Altshuler D, Daly MJ (2006). Evaluating and improving power in whole-genome association studies using fixed marker sets. Nat Genet.

